# Tobacco control measures in COVID-19 recovery: an opportune time to restore equity and planetary health

**DOI:** 10.1265/ehpm.21-00027

**Published:** 2022-03-31

**Authors:** Daniel Tzu-Hsuan Chen

**Affiliations:** Public Health Policy Evaluation Unit, School of Public Health, Imperial College London, United Kingdom

**Keywords:** Tobacco control, Climate change, Sustainability, Planetary health, Environmental health

## Abstract

Tobacco intersects with the COVID-19 pandemic not only in terms of health consequences, but also environmental change and planetary health. Tobacco use exacerbates inequalities, causes catastrophic environmental degradation and climate change and adds burdens to COVID-19-related mortality, which are major challenges to recovery from the COVID-19 pandemic. However, the pandemic has provided a chance to combat tobacco use and accelerate efforts to alleviate these challenges in response. The MPOWER measures introduced by the World Health Organization Framework Convention on Tobacco Control (WHO FCTC) can play a crucial role in COVID-19 recovery to fight tobacco use and contribute to sustainable and equitable development. To accelerate recovery, it is critical to call for actions for governments and policy-makers to strengthen synergies and coordinate policy actions emphasising tobacco control and cessation across equity, public health, and climate actions as global authorities pledge to achieve the Sustainable Development Goals (SDGs) and net zero emissions targets as part of the Climate Change Conference 2021 (COP26).

## Introduction

The global pandemic has significant and widespread impacts not only on the health of billions of people worldwide but also on public health systems, economies, and the environment. Globally, COVID-19 has caused over 6 million deaths; at the same time, tobacco kills more than 8 million people each year [1].

Tobacco intersects with COVID-19 not only in terms of health consequences, since it increases the risks of COVID-19 transmission, and puts patients at higher risks of severe illness when hospitalized [2], but also in terms of environmental change and planetary health [3]. The global tobacco supply contributes significantly to deforestation and pollution of the natural ecosystems. The damage is compounded by tobacco consumption and its resultant waste, which leaves a significant carbon footprint on the environment, undermining the planet’s ecological stability and intensifying climate change. Environmental factors such as pollution, temperature, and humidity have been linked to the spread and risks of COVID-19 [4]. The environmental impact caused by tobacco may well increase the danger of the pandemic.

COVID-19 recovery should be a concerted effort to strengthen public health systems, economies, and equitable societies in order to accelerate progress towards achieving the United Nations (UN) Sustainable Development Goals (SDGs). However, tobacco use is a major roadblock to key developments and an obstacle to recovery.

## The challenges in COVID-19 recovery

There is strong evidence that tobacco use increases the risks of mortality and disease severity among COVID-19 patients. Ecological studies have explored the relationship between COVID-19 hospitalisation and mortality with smoking [5, 6]. Countries and provinces with a higher age-standardized prevalence of tobacco smoking had a higher likelihood of COVID-19 death rates [6]. As part of the global COVID-19 recovery and to further avoid smoking-related burden on the pandemic, public health efforts during the pandemic should assist tobacco users in quitting and provide cessation support to tobacco users.

Tobacco use also exacerbates inequalities, increases poverty, and damages planetary health. The annual number of individuals globally who are in poverty because of the health costs of tobacco-related diseases is comparable to the number of people who have become impoverished due to the COVID-19 pandemic [7]. Moreover, tobacco consumption and production generate tonnes of toxic waste, releasing thousands of chemicals into the planet’s air, water, and soil and emitting approximately 84 million tonnes of greenhouse gases (GHG), which accounts for approximately 0.2% of the world’s total GHG emissions, nearly as much as an entire country’s emissions towards climate change [3]. However, the most disadvantaged populations, who are least accountable for the environmental damage created by climate change and tobacco use, are the most impacted by its repercussions.

Tobacco contributes to all of the aforementioned global challenges. Reducing and ultimately eliminating tobacco production and consumption remains one of the global priorities. Nonetheless, it is frequently overlooked and rarely appears on the agendas of government plans. Tobacco control measures should be explicitly acknowledged as an integral part of strategies in environmental, equity, and health-focused policy responses as joint solutions to interconnected problems for a COVID-19 sustainable recovery. Public health actions to promote smoking cessation and decrease tobacco use should be implemented at all levels as a means of reducing disease burden, particularly in countries with a higher prevalence of use as preventative measures to combat the pandemic. Furthermore, in response to the goals set out at the United Nations Climate Change Conference 2021 (COP26) [8], it is important to deliver synergized policy action emphasising tobacco control within climate actions as authorities pledge to move toward net zero emissions targets and restore ecosystems.

## Tobacco control measures in boosting a sustainable and equitable recovery for a healthier planet

Strengthened tobacco control measures reduce global tobacco use and protect the planet and people from the health, environmental, and social consequences of its use. Key MPOWER measures from the World Health Organization Framework Convention on Tobacco Control (WHO FCTC) [1] can serve as entry points to boost sustainable and equitable COVID-19 recovery — *Monitor* tobacco use (article 20); *Protection* from tobacco smoke (article 8); *Offer* help for tobacco cessation (article 14); *Warn* about the dangers of tobacco (article 11); and *Raise* tobacco taxes (article 6).

Monitoring and surveillance of tobacco use is a crucial tobacco control measure. However, there is still lack of consistent monitoring on tobacco product use in large scale COVID-19-related surveys. Integration of COVID-19 questions and records of exposures to smoking in national and international studies is essential to advance our understanding of the impact of tobacco use on COVID-19 and build up reliable epidemiological evidence to tackle the cooccurrence of the smoking and COVID-19 pandemic.

Tobacco smoke compromises indoor air quality, contributes negatively to climate change and the environment, and has been linked to over a million deaths worldwide [9]. Second-hand smoke (SHS) exposure is an established risk factor for cardiovascular and chronic lung diseases and is also a risk factor for COVID-19, either via its impact on non-communicable diseases (NCDs) or through its involvement in COVID-19 transmission. During the pandemic, the extra time spent indoors may have exposed many to higher levels of SHS and exposure to infectious diseases. Strengthened smoke-free laws to protect exposure to SHS and tobacco smoke not only assist in reducing health risks but also susceptibility to respiratory tract infections [2]. At the planetary level, it improves air quality and reduces health risks that are attributable to SHS and COVID-19 transmissions associated with environmental factors.

With regards to smoking cessation, the COVID-19 pandemic is a unique window of opportunity to promote and support tobacco cessation services. There is robust evidence that smoking cessation programmes are crucial for reducing tobacco use [10], and they are undoubtedly one of the most important tobacco control measures driven by the pandemic. However, unequal access to cessation programmes persists as a barrier to reaching and helping disadvantaged smokers in their efforts to quit smoking — approximately 60% of tobacco users worldwide have expressed an interest in quitting, and many of them do not have access to services [1]. Currently, only 23 countries offer comprehensive cessation programs, including cost coverage to help people stop, accounting for only 32% of the world’s population [1]. With an increasing number of smokers willing to quit post-COVID-19 [10], there is a need for a coordinated response to the tobacco epidemic at the country level, which should include improved access to smoking cessation aids and programs. Governments, especially developing countries and nations with high prevalence of tobacco use, need to invest in effective cessation interventions and improve the accessibility of services to reduce tobacco consumption and raise awareness about the dangers of tobacco to reduce burdens on COVID-19-related mortality and gaps in inequality as part of the recovery plan.

Health warnings on tobacco packaging, enforced bans on promotion and advertising, and raising tobacco taxes are proven effective WHO MPOWER demand-reduction measures to reduce tobacco use at the population level [1]. Evidence has shown that strengthened implementation of these key measures results in dose–response effects in lowering tobacco use prevalence and improved levels of compliance with comprehensive smoke-free policies [9]. Particularly, to tackle the global pandemic, global authorities should emphasise such measures to reduce overall tobacco use.

An increase in tobacco taxation is deemed one of the most effective measures to curb tobacco consumption [1] and at the same time, provides governments with much needed financial resources for COVID-19 response and recovery efforts. Unfortunately, in times of crisis, such actions are sometimes perceived as an extra burden. Many countries have redeployed resources to combat the epidemic rather than strengthen tobacco control measures, which is a huge opportunity that has been missed. In the face of the pandemic, it is critical to improve policy coordination across the fiscal and public health sectors in terms of tobacco taxation and consumption reduction. However, India, South Africa, and Russia are the only countries that have increased tobacco taxes to support governments in their COVID-19 response and recovery efforts [1]. Echoing the examples of these nations, it is imperative to urge governments to strengthen vital strategies for stronger tobacco control policies in this unprecedented time.

The collective effects of strengthening all MPOWER policies as part of the post-COVID-19 recovery plan not only improve population health but also alleviate poverty, restore equality, and limit climate change and environmental degradation threats. Reducing tobacco use protects against the future prevalence of tobacco-related diseases and redirects spending towards health care and other investments that could lift the disadvantaged out of poverty [1]. Moreover, tobacco control and action against climate change are mutually reinforcing — tobacco control provides a comprehensive approach to the enhancement and conservation of biomass, forests, oceans, and other ecosystems. The reduction in tobacco use will lead to fewer littered cigarette wastes, less chemical contamination of water, and less air pollution from SHS and tobacco smoke.

## Counteraction against tobacco industry interference at the time of COVID-19

The WHO FCTC also calls for efforts to reduce the supply of tobacco and the implementation of measures to help prevent tobacco industry interference (article 5.3). Such measures may play an important role in sustainable recovery post-COVID-19. The tobacco industry took advantage of public concern about the global pandemic to seek partnerships with governments and institutions as part of its public relations strategy to improve its image and legitimacy, as well as by giving out gifts and promotional personal protective items to the public, sending misleading messages that their products are safe and benign. Governments need to remain alert to this so-called corporate social responsibility (CSR) by prohibiting and regulating industries’ interference during and beyond the COVID-19 pandemic to counteract their invested commercial interests.

Finally, the COVID-19 pandemic has resulted in major employment losses and a sharp decline in national economies globally. However, for nations that depend heavily on tobacco farming, this might be a pivotal moment to transition away from tobacco production towards activities that are safer for people and the planet, with tobacco control measures providing support for alternative economic livelihoods for tobacco farmers (article 17). This is especially pertinent in developing countries such as China, where tobacco farming has resulted in widespread deforestation.

## Conclusions

The MPOWER measures can play a vital role in boosting sustainable and equitable recovery. Deaths due to tobacco and environmental factors are fully avoidable and would only cost a fraction of the cost of the pandemic. Global health authorities must act collectively now to call for governments and policy-makers to include tobacco control and advance implementations of key tobacco control measures in COVID-19 recovery plans, as this appears as an opportune time to combat tobacco use and accelerate progress towards sustainable planetary health while restoring equitable societies.

Governments must provide means for tobacco users to quit to reduce disease burden associated with tobacco use and COVID-19 and strengthen policies to counteract tobacco industry interference. Climate actions should include environmental protection from tobacco use and its resultant waste, as well as initiatives to shift tobacco growing to alternative livelihoods to combat global warming and climate change.
